# A Genome-Wide Screen Identifies Yeast Genes Required for Tolerance to Technical Toxaphene, an Organochlorinated Pesticide Mixture

**DOI:** 10.1371/journal.pone.0081253

**Published:** 2013-11-18

**Authors:** Brandon D. Gaytán, Alex V. Loguinov, Xenia Peñate, Jan-Michael Lerot, Sebastián Chávez, Nancy D. Denslow, Chris D. Vulpe

**Affiliations:** 1 Department of Nutritional Science and Toxicology, University of California, Berkeley, Berkeley, California, United States of America; 2 Departamento de Genética, Universidad de Sevilla and Instituto de Biomedicina de Sevilla, Hospital Universitario Virgen del Rocío/CSIC/Universidad de Sevilla, Seville, Spain; 3 Department of Physiological Sciences and Center for Environmental and Human Toxicology, University of Florida, Gainesville, Florida, United States of America; CSIR-Central Drug Research Institute, India

## Abstract

Exposure to toxaphene, an environmentally persistent mixture of chlorinated terpenes previously utilized as an insecticide, has been associated with various cancers and diseases such as amyotrophic lateral sclerosis. Nevertheless, the cellular and molecular mechanisms responsible for these toxic effects have not been established. In this study, we used a functional approach in the model eukaryote *Saccharomyces cerevisiae* to demonstrate that toxaphene affects yeast mutants defective in (1) processes associated with transcription elongation and (2) nutrient utilization. Synergistic growth defects are observed upon exposure to both toxaphene and the known transcription elongation inhibitor mycophenolic acid (MPA). However, unlike MPA, toxaphene does not deplete nucleotides and additionally has no detectable effect on transcription elongation. Many of the yeast genes identified in this study have human homologs, warranting further investigations into the potentially conserved mechanisms of toxaphene toxicity.

## Introduction

**Figure 1:**
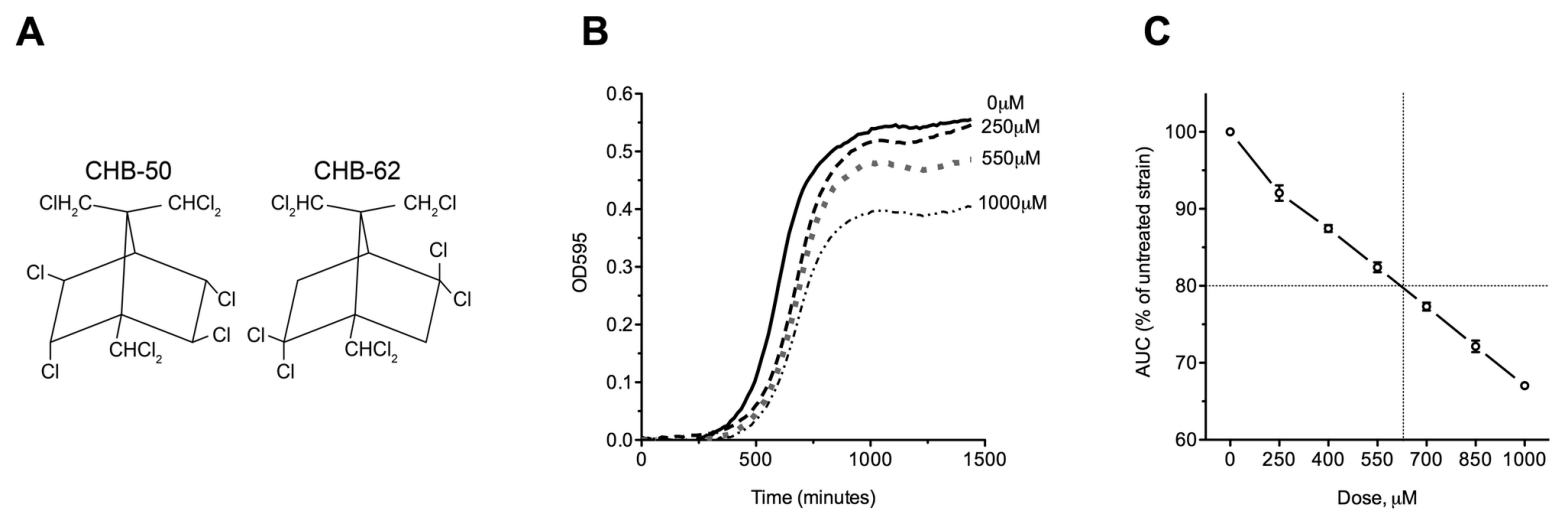
Determining the toxaphene IC20 for functional profiling. (**A**) The structure of two chlorinated congeners present in the toxaphene technical mixture. (**B**) Representative growth curves in YPD media for the BY4743 wild-type strain treated with toxaphene. For clarity, only the 250, 550, and 1000μM toxaphene doses are shown, although additional curves were performed at 400, 700, and 850μM. (**C**) The area under the curve (AUC) was calculated at each dose for three independent experiments, expressed as the mean and SE, and plotted as a percentage of the untreated control. The toxaphene IC20 was determined to be 640μM, as indicated by the dashed lines.

Toxaphene is a complex mixture of polychlorinated camphenes and bornanes primarily used to control insects on cotton during the 1960-80s (Figure 1A) [1]. After the ban of DDT in 1972, toxaphene became the most heavily applied pesticide in the United States, but all registered uses were cancelled by the U.S. Environmental Protection Agency (EPA) in 1989 over concerns related to its toxicity and persistence [2]. Today, toxaphene remains a problematic environmental contaminant, ranking 32^nd^ on the Agency for Toxic Substances and Disease Registry (ATSDR) Priority List of Hazardous Substances, a list of compounds that possibly threaten human health via their toxicity and possibility for exposure at EPA National Priorities List hazardous waste sites. Toxaphene's most persistent congeners and degradation products have been detected in water, air, and sediment, and are known to bioaccumulate in wildlife and humans [1]. Animal studies have deemed toxaphene a neuro-, nephro-, immuno-, and hepatotoxicant, an endocrine disruptor, and a carcinogen, with the International Agency for Research on Cancer (IARC) classifying toxaphene as Group 2B (possibly carcinogenic to humans) [1,3]. Epidemiological analyses have linked toxaphene to leukemia [4], non-Hodgkin's lymphoma [5], melanoma [6], and more recently, amyotrophic lateral sclerosis [7]. However, the cellular and molecular processes that toxaphene perturbs to result in these toxicities and disease states remain unclear.

The eukaryotic yeast *Saccharomyces cerevisiae* is a valuable model in which to conduct toxicological studies. First, basic cellular processes, along with metabolic and signaling pathways, are conserved to higher organisms. Second, a close human homolog has been identified for a significant portion of yeast genes, with several hundred of the conserved genes linked to disease in humans [8]. Finally, the availability of deletion mutant collections, overexpression libraries, and genetic and physical interaction data provide unmatched resources for inquiries into potential cellular and molecular mechanisms of toxicity. With the deletion mutant collection [9], functional toxicogenomic analyses (also known as functional profiling) can be conducted by examining, in parallel, the sensitivity or resistance of each mutant strain to a compound of interest. Such investigations, through the identification of yeast genes required for chemical tolerance, have discovered molecular mechanisms for numerous drugs and toxicants [10,11], with several studies confirming results in human cells [12-14].

Here we present the results of a functional screen devised to identify yeast genes necessary for growth in the presence of toxaphene. It is the first known genome-wide study in any organism to examine the genetic requirements for toxaphene tolerance. Our results demonstrate that similar to the known transcription elongation inhibitors mycophenolic acid (MPA) and 6-azauracil (6-AU), mutants defective in processes linked to transcription elongation are sensitive to toxaphene. While toxaphene does display synergism with MPA, it apparently exhibits a mechanism of action distinct from that of MPA/6-AU and additionally does not appear to directly affect transcription elongation. Many yeast genes required for toxaphene resistance have human homologs that may play a role in human toxicity.

## Materials and Methods

### Yeast strains and culture

The diploid non-essential yeast deletion strains used for functional profiling and confirmation analyses were of the BY4743 background (*MATa/MATα his3Δ1/his3Δ1 leu2Δ0/leu2Δ0 lys2Δ0/LYS2 MET15/met15Δ0 ura3Δ0/ura3Δ0*, Life Technologies). Yeast growth was performed in liquid rich media (1% yeast extract, 2% peptone, 2% dextrose, YPD) at 30°C with shaking at 200 revolutions per minute (rpm). For the elongation assays, the *GAL1-YLR454* strain [15] was grown in rich media containing galactose (1% yeast extract, 2% peptone, 2% galactose, YPGal).

### Dose-finding and growth curve assays

Dose-finding and growth curves were performed as in [16]. Toxaphene and MPA (Sigma Aldrich) solutions were prepared in dimethylsulfoxide (DMSO) and added to the desired final concentrations (1% or less by volume) with at least three technical replicates per dose. The area means and standard error (SE) for the growth of each strain (as measured by the area under the curve) was derived from three independent cultures.

### Functional profiling of the yeast genome

The functional screen and differential strain sensitivity analysis (DSSA) were performed as previously described [17]. Briefly, pools of deletion mutants (*n* = 4607) were cultured for 15 generations in YPD at various toxaphene concentrations. Following extraction of genomic DNA with the YDER kit (Pierce Biotechnology), DNA sequences unique to each strain were amplified by PCR and subsequently hybridized to TAG4 arrays (Affymetrix). Arrays were incubated overnight, stained, and scanned at 560 nm with a GeneChip Scanner (Affymetrix). Data files are available at the Gene Expression Omnibus database (http://www.ncbi.nlm.nih.gov/geo/).

### Over-enrichment and network mapping analyses

The Functional Specification (FunSpec) resource [18] identified significantly overrepresented Gene Ontology (GO) and MIPS (Munich Information Center for Protein Sequences) categories within the DSSA data using a *p* value cutoff of 0.002 and Bonferroni correction. Gene interaction networks were generated in Cytoscape [19] by mapping fitness scores for toxaphene-sensitive strains onto the BioGrid *Saccharomyces cerevisiae* functional interaction network. The jActiveModules Cytoscape plugin identified sub-networks enriched with fitness data, within which the BiNGO plugin discovered overrepresented GO categories. 

### Analysis of relative strain growth by flow cytometry

Analyses of relative strain growth were performed as described [16]. Briefly, green fluorescent protein (GFP)-tagged wild-type and untagged deletion strains were mixed in approximately equal numbers and cultured for 24 hours at 30°C in the presence of toxaphene. At least 20,000 cells were analyzed at T=0 and T=24 hours using a FACSCalibur flow cytometer. A ratio of growth was calculated for untagged cells in treated versus untreated samples using the percentages of wild-type GFP and untagged mutant cells present. Statistically significant differences between the means of three independent DMSO-treated and toxaphene-treated cultures were determined with Student’s *t*-test. 

### Analyses of transcription elongation

Gene Length Accumulation of mRNA (GLAM) assays were performed as described [20]. Chromatin immunoprecipitation experiments were performed as described [21], except mouse monoclonal anti-Rpb3 (1Y26, Abcam) antibody was used. Immunoprecipitated DNA was measured by quantitative PCR in a LightCycler (Roche), using the primers listed in Table S1. Normalizations required for RNA polymerase II processivity comparisons were applied as described [15]. 

## Results

### Functional profiling in yeast identifies genes required for toxaphene tolerance

The IC20, the concentration at which growth is inhibited by 20%, is a dose frequently utilized in functional screens, as it elicits a response without being overly toxic [17]. After performing growth curve analyses for wild-type yeast treated with increasing concentrations of toxaphene (Figure 1B), the toxaphene IC20 was calculated as 640μM (Figure 1C). Pools of yeast homozygous diploid deletion mutants (*n* = 4607) were grown for 15 generations at the toxaphene IC20 (640μM), 50% IC20 (320μM), and 25% IC20 (160μM) to identify genes important for growth in toxaphene. DSSA deemed 130 strains sensitive and 542 strains resistant to at least one dose of toxaphene (Table S2). Sensitive strains were the focus of this study, with the top 30 displaying growth defects shown in Table 1. 

**Table 1:** Fitness scores for the top 30 deletion strains identified as significantly sensitive to toxaphene after 15 generations of growth.

	**Log_2_ value**s				
**Deleted Gene**	**25% IC20 160μM**	**50% IC20 320μM**	**100%IC20 640μM**	**Function of deleted gene**	**Confirmed**
***PDR5***	-6.30	-5.80	-6.05	Plasma membrane multidrug transporter	S
***RPN4***	-3.15	-3.05	-2.55	Transcription factor; stimulates expression of proteasome genes	S
***DAL81***	-3.10	-3.50	-3.00	Positive regulator of genes in nitrogen degradation pathways	S
***TRP4***	-2.90	-4.50	-4.70	Anthranilate phosphoribosyl transferase for tryptophan biosynthesis	S
***SWF1***	-2.90	-3.20	-3.00	Palmitoyltransferase; acts on various SNAREs	NS
***URE2***	-2.75	-2.45	-2.95	Nitrogen catabolite repression transcriptional regulator	S
***STP1***	-2.50	-3.10	-3.00	Transcription factor for amino acid permease genes	S
***YGR153W***	-2.50	-2.60	-2.60	Putative protein of unknown function	
***YDR008C***	-2.30	-2.80	-3.10	Dubious ORF; partially overlaps *TRP1* tryptophan biosynthetic enzyme	
***SEC 66***	-2.30	-2.60	-2.40	Subunit of Sec63 complex; involved in ER protein translocation	
***YEL045C***	-2.20	-2.60	-2.40	Dubious ORF	
***PDR1***	-2.15	-2.25	-2.70	Transcriptional regulator of multidrug resistance genes	S
***SPT4***	-2.00	-1.85	-1.95	Regulates Pol I and Pol II transcription	S
***IRS4***	-1.75	-2.50	-2.00	Regulates phosphatidylinositol 4,5-bisphosphate levels and autophagy	S
***YKL077W***	-1.75	-1.45	-1.20	Putative protein of unknown function	
***THP2***	-1.65	-2.00	-2.50	Subunit of the THO complex; involved in transcription elongation	S
***YDR203W***	-1.60	-1.90	-2.20	Dubious ORF; partially overlaps *RAV2*	
***ICE2***	-1.50	-1.50	-2.20	Integral ER membrane protein	
***TKL1***	-1.50	-1.40	-1.70	Transketolase in the pentose phosphate pathway	S
***COG7***	-1.50	-1.40	-1.40	Component of the COG golgi transport complex	
***COX20***	-1.45	-1.85	-2.05	Mitochondrial inner membrane protein	
***IMG2***	-1.40	-1.70	-2.60	Mitochondrial ribosomal protein of the large subunit	
***YDR455C***	-1.40	-1.50	-1.30	Dubious ORF; partially overlaps *NHX1*	
***URA2***	-1.30	-1.40	-1.55	Involved in *de novo* biosynthesis of pyrimidines	NS
***SIW14***	-1.30	-1.30	-1.30	Tyrosine phosphatase	
***GYP1***	-1.25	-1.55	-1.50	cis-golgi GTPase-activating protein (GAP) for the Rab family	NS
***YSP1***	-1.25	-1.50	-1.75	Mitochondrial protein; potential role in programmed cell death	S
***YJL120W***	-1.25	-1.30	-1.35	Dubious ORF; partially overlaps RPE1	
***CUE1***	-1.20	-1.00	-1.15	ER membrane protein; regulates ubiquitination	
***PBP1***	-1.20	-0.90	-1.10	Component of glucose deprivation induced stress granules	

Fitness scores, defined as the normalized log_2_ ratio of strain growth in the presence versus absence of toxaphene, quantify the requirement of a gene for growth. The confirmed column indicates whether the strain was confirmed as sensitive (S) or not sensitive (NS- a false positive) by growth curve assays. The DSSA used to identify altered strain growth is semi-quantitative, as variations in individual strain growth along with initial deletion pool strain counts can impact relative log_2_ ratios. Table S2 contains a list of all mutants displaying altered growth in the functional screen.

### Overrepresentation analyses identify biological attributes needed for toxaphene resistance

**Figure 2:**
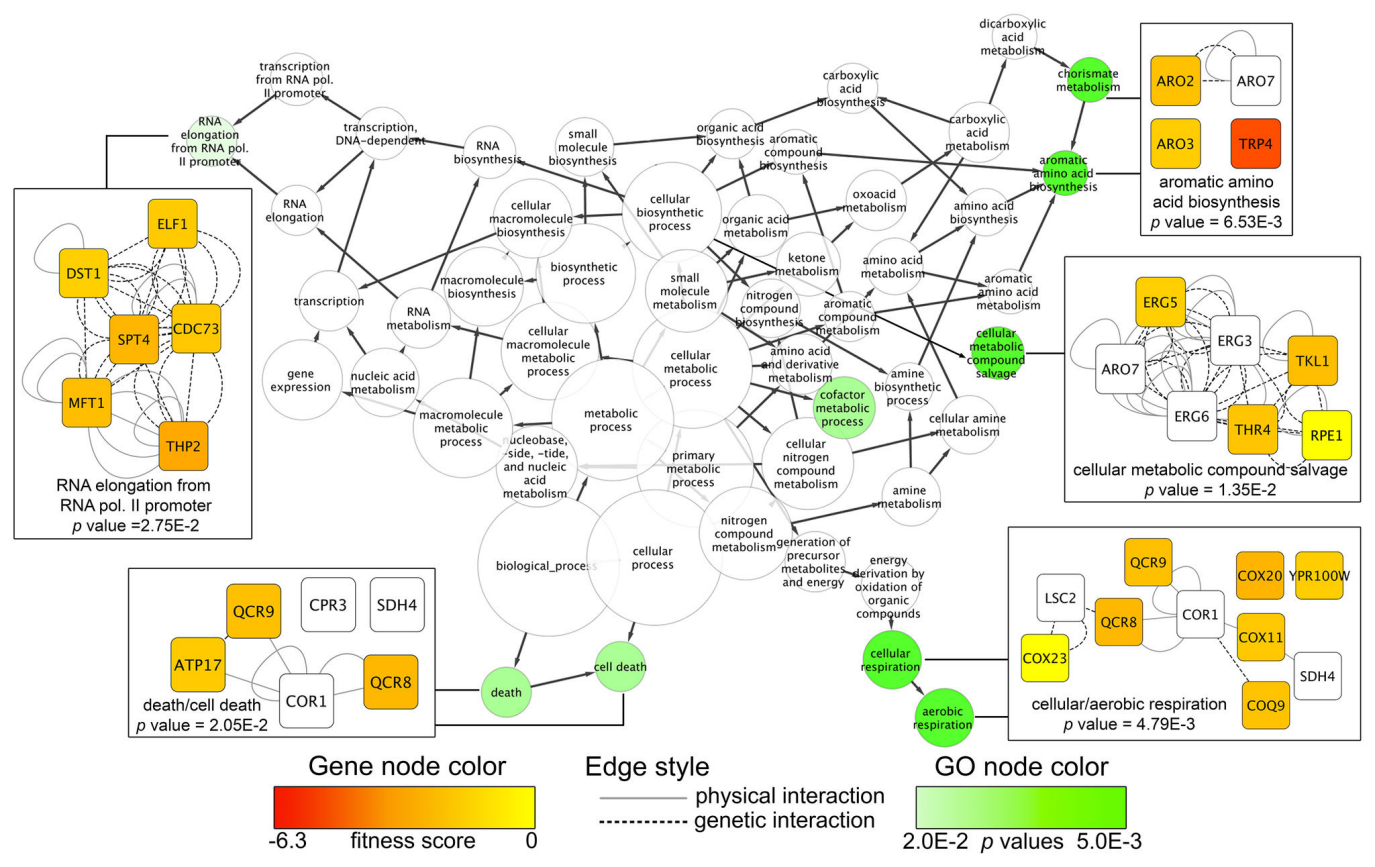
Biological attributes required for toxaphene tolerance are identified by network mapping. Cytoscape was used to map fitness scores (the ratio of the log_2_ hybridization signals between DMSO and toxaphene exposures) for toxaphene-sensitive strains onto the *Saccharomyces cerevisiae* BioGRID interaction dataset. A subnetwork (*n* = 104) containing genetic and physical interactions between the sensitive, non-sensitive, and essential genes was created and significantly overrepresented (*p* value cutoff of 0.03) GO categories were identified. The green node color corresponds to the GO *p*-value while the node size correlates to the number of genes in the category. Edge arrows indicate hierarchy of GO terms. Networks for various GO categories are shown, where node color corresponds to deletion strain fitness score and edge defines the type of interaction between the genes.

All toxaphene-sensitive strains identified by DSSA (*n* = 122; Table S2) were analyzed with FunSpec for significantly overrepresented biological attributes at a corrected *p* value of 0.002 (Table 2). Transcription elongation and aerobic respiration were enriched in both GO and MIPS categories, indicating that mutants lacking genes associated with these processes are sensitive to toxaphene. Other overrepresented GO classifications included mitochondrial respiratory chain complex IV biogenesis and aromatic amino acid biosynthesis. To supplement the FunSpec evaluation, network mapping was performed with Cytoscape to identify additional attributes required for toxaphene tolerance. The BiNGO plugin revealed that transcription elongation, aerobic respiration, and aromatic amino acid biosynthesis were overrepresented within the network data (Figure 2). Other categories uncovered were cell death and metabolic salvage. 

**Table 2:** Genes required for toxaphene tolerance and their associated GO or MIPS categories.

**GO Biological Process**	***p* value**	**Genes identified**	**k^a^**	**f^b^**
mitochondrial respiratory chain complex IV biogenesis (GO:0097034)	3.14E-004	*PET122, PET54, PET494*	3	8
regulation of ER to Golgi vesicle-mediated transport (GO:0060628)	3.33E-004	*UBP3, BRE5*	2	2
transcription elongation from RNA polymerase II promoter (GO:0006368)	4.21E-004	*DST1, SPT4, THP2, ELF1, CDC73, MFT1*	6	54
Group I intron splicing (GO:0000372)	6.56E-004	*PET54, CBP2, MRS1*	3	10
aerobic respiration (GO:0009060)	8.16E-004	*COX20, QCR9, QCR8, COQ9, COX11, MRPL51*	6	61
response to arsenic-containing substance (GO:0046685)	8.90E-004	*RPN4, GET3, TIM18*	3	11
heme a biosynthetic process (GO:0006784)	9.87E-004	*COX15, COX10*	2	3
ribophagy (GO:0034517)	9.87E-004	*UBP3, BRE5*	2	3
aromatic amino acid family biosynthetic process (GO:0009073)	1.17E-003	*ARO3, TRP4, ARO2*	3	12
**MIPS Functional Classification**	***p* value**	***Genes identified***	**k^a^**	**f^b^**
transcription elongation (11.02.03.01.04)	1.69E-005	*DST1, SPT4, THP2, ELF1, CDC73, MFT1*	6	31
aerobic respiration [02.13.03)	4.85E-004	*COX20, QCR9, COX23, QCR8, COQ9, COX11, MRPL51*	7	77

Strains exhibiting sensitivity to three doses of toxaphene in the functional screen were entered into FunSpec and analyzed for overrepresented biological attributes (see Materials and Methods section). ^a^ Number of genes in category identified as sensitive to toxaphene. ^b^ Number of genes in GO or MIPS category.

### Mutants defective in transcription elongation associated processes are sensitive to toxaphene

**Figure 3:**
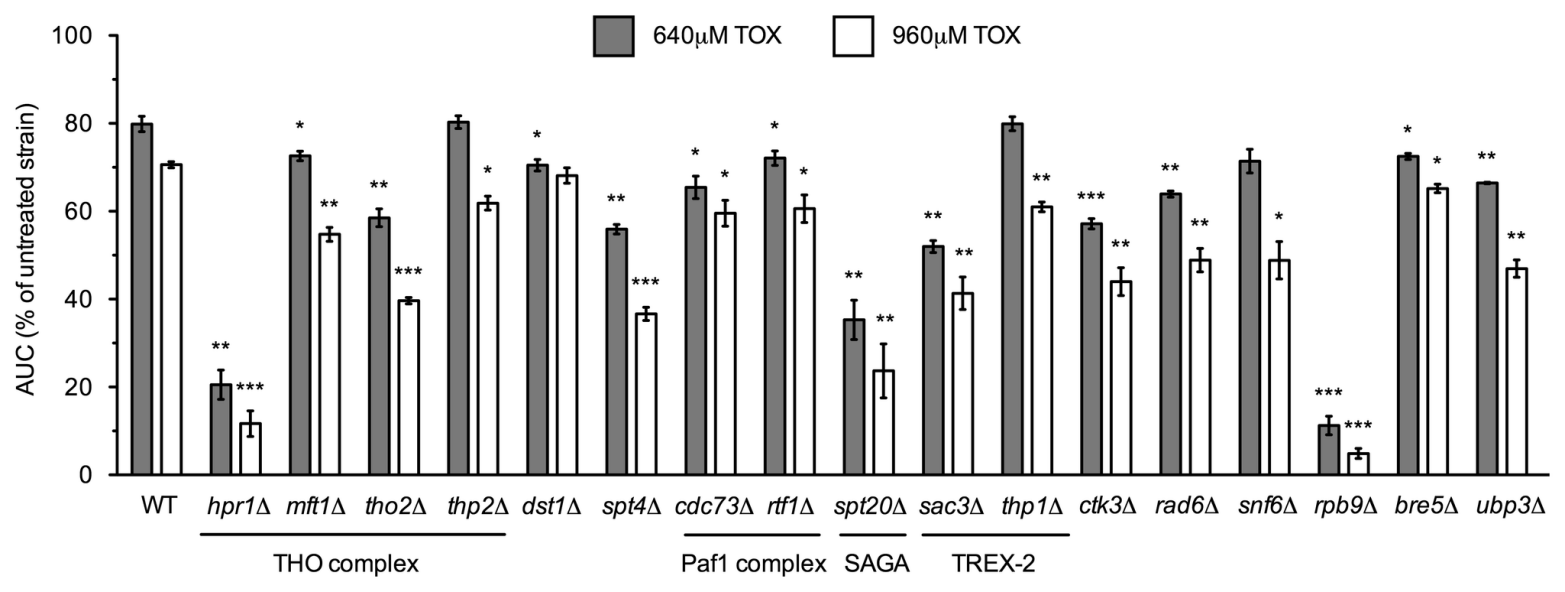
Transcription elongation mutants are sensitive to toxaphene. Growth curves for three independent cultures were obtained for the indicated strains and toxaphene concentrations. Mutants with known defects in transcription elongation are sensitive to toxaphene, including members of the THO, Paf1p, SAGA, and TREX-2 complexes. Additional mutants lacking genes implicated in transcription elongation exhibit sensitivity as well. The AUC was calculated for each curve and is shown as a percentage of the untreated strain. Statistical significance between the wild-type and mutant strains was calculated with Student's *t*-test, where ***p<0.001, **p<0.01, and *p<0.05.

FunSpec and Cytoscape analyses indicated enrichment of transcription elongation mutants within the functional profiling data. Accordingly, growth curves were obtained for the transcription elongation mutants identified in the screen and compared to the BY4743 wild-type strain, with five out of the six (*cdc73*Δ, *dst1*Δ , *mft1*Δ, *thp2*Δ, and *spt4*Δ, but not *elf1*Δ) confirmed as sensitive to toxaphene (Figure 3). Considering two of these mutants (*mft1*Δ and *thp2*Δ) lacked components of THO, a complex required for efficient transcription elongation [22] and mRNA export [23], we tested strains harboring deletions of the two additional THO subunits (*hpr1*Δ and *tho2*Δ), finding growth defects (Figure 3). Many of these confirmed strains are also sensitive to the transcription elongation inhibitors MPA and 6-AU (Table S3) [24], therefore, we examined whether toxaphene affected a set of MPA- or 6-AU-sensitive mutants known to exhibit transcription elongation defects. Indeed, strains lacking subunits of the SAGA histone acetyltransferase (*spt20*Δ), the Paf1p complex (*rtf1*Δ), and the TREX-2 transcriptional export machinery (*sac3*Δ and *thp1*Δ) were sensitive to toxaphene (Figure 3). Additionally, a gene encoding an RNA polymerase II subunit (*RPB9*), along with genes implicated in RNA polymerase II activation (*CTK3*, *RAD6*, and *SNF6*) or reactivation through deubiquitylation (*BRE5* and *UBP3*) were required for toxaphene tolerance (Figure 3).

### Nitrogen utilization and aromatic amino acid biosynthesis mutants are sensitive to toxaphene

**Figure 4:**
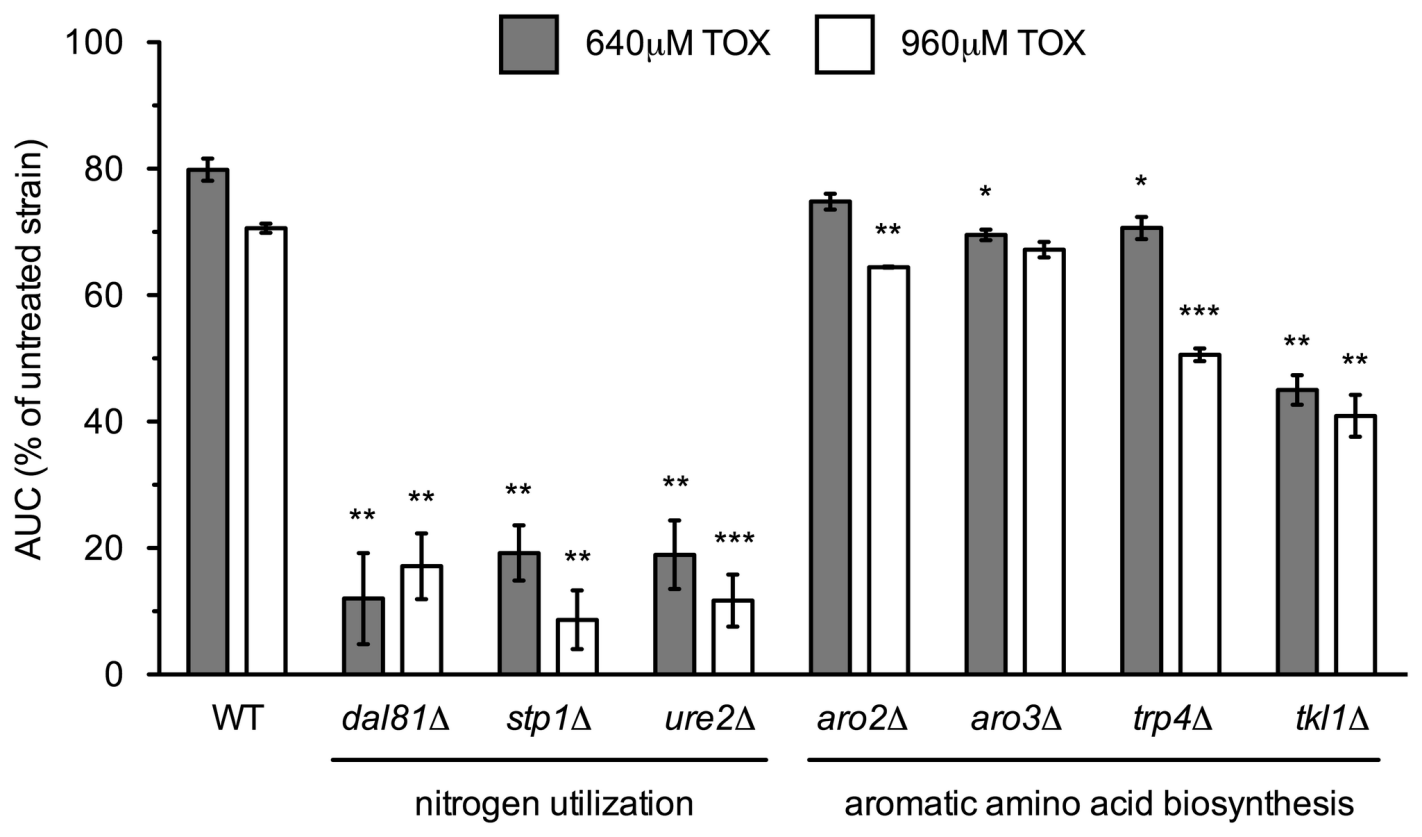
Nitrogen utilization and aromatic amino acid synthesis genes are required for toxaphene tolerance. The AUC was calculated for strains treated with 640μM or 960μM toxaphene and expressed as a percentage of the AUC for the untreated strain. Bars signify the mean and SE for three independent cultures.

The *dal81*Δ, *stp1*Δ, and *ure2*Δ nitrogen utilization mutants identified in the functional screen (Table 1) were confirmed by growth curve analyses to exhibit severe growth defects in presence of toxaphene (Figure 4). For unknown reasons, the growth of these mutants is also hindered by the transcription elongation inhibitors MPA and 6-AU [24,25]. Additionally, in agreement with enrichment analyses (Figure 2 and Table 2), mutants deficient in aromatic amino acid biosynthesis were toxaphene-sensitive, with the *TRP4* tryptophan biosynthesis gene implicated as the pathway component furthest downstream (Figure 4). However, neither tryptophan supplementation of YPD media nor titration into defined media altered growth of the BY4743 wild-type strain in toxaphene (data not shown).

### Toxaphene exhibits synergy with MPA, but its mechanism of action is not similar

**Figure 5:**
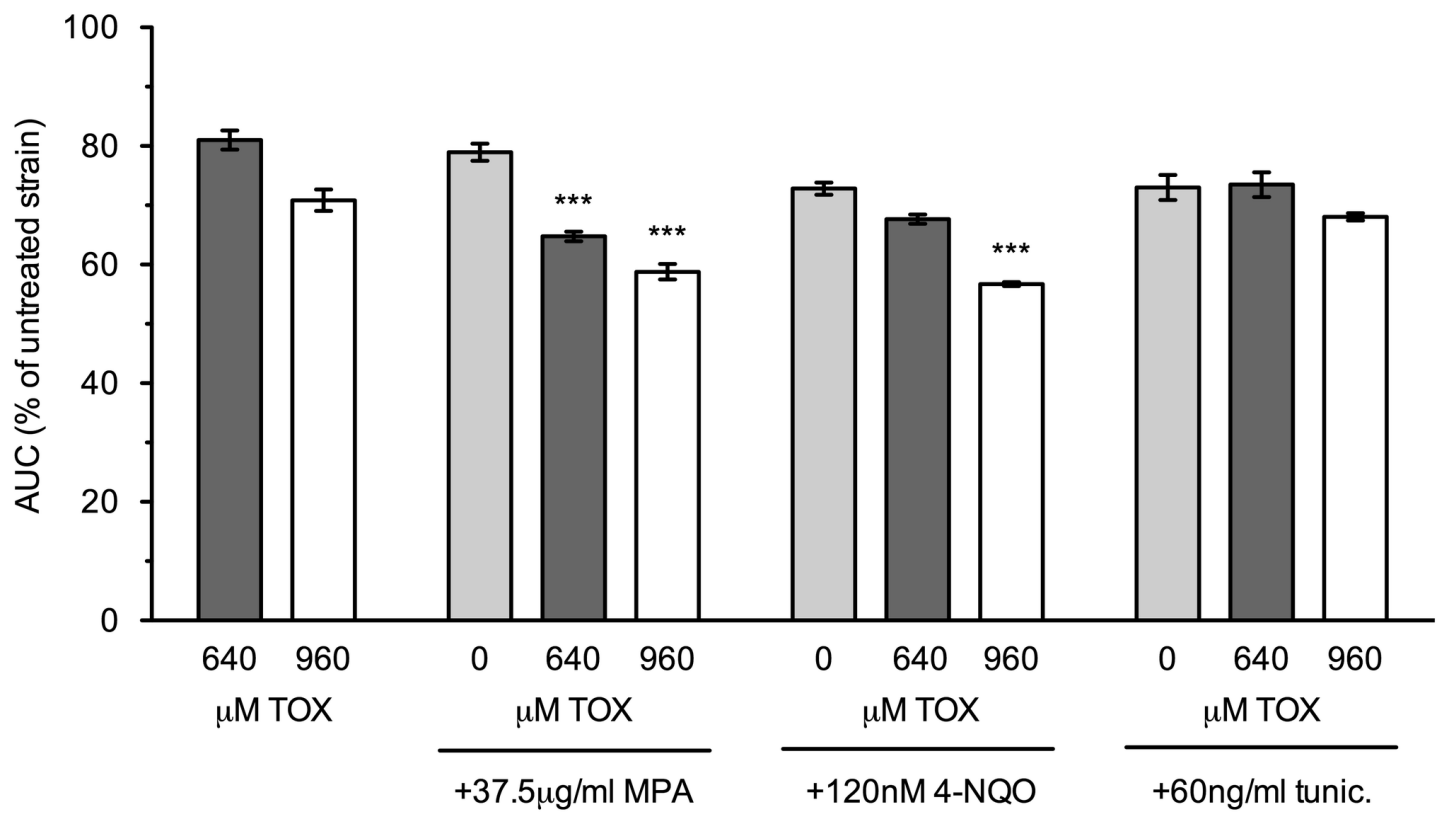
Toxaphene exhibits synergy with MPA and 4-NQO. Growth curve assays were performed for three independent cultures of the BY4743 wild-type strain with the indicated compounds. Both MPA (a transcription elongation inhibitor) and 4-NQO (a genotoxicant) displayed synergy with toxaphene, while tunicamycin (an endoplasmic reticulum stressor) did not. Statistical significance was determined with one-way ANOVA with a Tukey post-test. *** represents significance observed at *p*<0.001 between both toxaphene/toxaphene+synergist and synergist/toxaphene+synergist comparisons.

**Figure 6:**
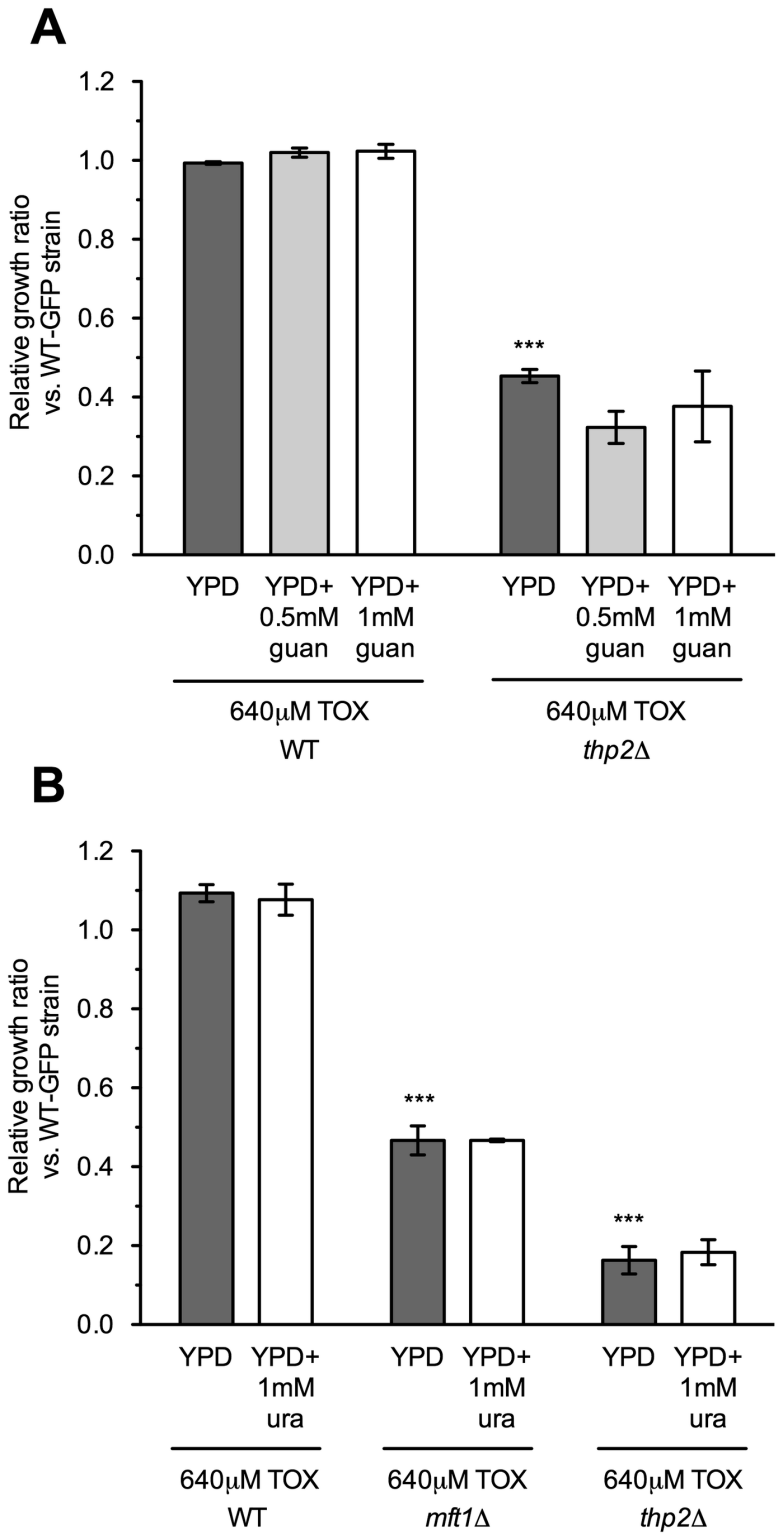
Neither guanine nor uracil rescues toxaphene sensitivity of transcription elongation mutants. Relative growth ratios (treatment vs. control) to a GFP-expressing wild-type strain were obtained for three independent cultures and the means and SEs are shown. One-way ANOVA followed by a Bonferroni post-test determined statistical significance. ***p<0.001 for wild-type/mutant comparisons. (**A**) The toxaphene sensitivity of the *thp2*Δ strain cannot be rescued by guanine. YPD media was supplemented with the indicated concentrations of guanine and toxaphene. (**B**) Uracil cannot reverse the toxaphene sensitivity of the *mft1*Δ or *thp2*Δ strains. Uracil and toxaphene were added to YPD media at the indicated concentrations. The impact of toxaphene on *mft1*Δ and *thp2*Δ was stronger in these relative growth experiments versus growth curve assays (see Figure 3). Most likely, this is due to the competitive nature of the relative growth assays, where even in control experiments, deletions with intrinsic growth defects are outcompeted by wild-type. Exposure to toxaphene likely magnified the relative growth defects between *mft1*Δ or *thp2*Δ and the wild-type strain in this assay.

Similarities between the mutant sensitivity profiles for toxaphene and the transcription elongation inhibitors MPA and 6-AU (Table S3) prompted us to examine whether the compounds shared a mechanism of action. Both MPA and 6-AU inhibit the guanosine monophosphate (GMP) synthesis enzyme inosine monophosphate dehydrogenase (*IMPDH*), which reduces ribonucleotide levels and increases dependence on transcription elongation factors for transcriptional efficiency [26,27]. As a first step, we assessed synergy between toxaphene and MPA by obtaining growth curves for each condition as well as the mixture. We also considered 4-nitroquinoline-N-oxide (4-NQO), a model carcinogen whose DNA adducts may be repaired by transcription-coupled nucleotide excision repair [25], and tunicamycin, an endoplasmic reticulum stressor [28]. Both MPA and 4-NQO displayed additive inhibitory growth effects with toxaphene, while the general stressor tunicamycin did not (Figure 5). Second, we examined whether guanine or uracil supplementation could reverse toxaphene sensitivity, as both can rescue the growth of transcription elongation mutants in the presence of either MPA or 6-AU [24,26]. A flow cytometry assay, in which relative growth of a mutant strain to a wild-type GFP-expressing strain was compared under the indicated conditions, confirmed that neither guanine nor uracil reversed toxaphene sensitivity of transcription elongation mutants (Figures 6A and B). Third, we tested a strain deleted for *IMD2*, the only yeast *IMPDH* homologue that provides resistance to MPA [29], for toxaphene sensitivity. Although hypersensitive to MPA, the *imd2*Δ strain did not display altered growth in toxaphene (data not shown). Finally, we found toxaphene did not affect strains deleted for *URA2* or *URA4*, enzymes involved in the *de novo* biosynthesis of pyrimidine ribonucleotides [30] (data not shown). Collectively, these data suggest that toxaphene's mechanism is not analogous to MPA/6-AU, i.e., it does not alter nucleotide pools or target the *IMPDH* family of enzymes.

### Toxaphene does not affect transcription elongation

**Figure 7:**
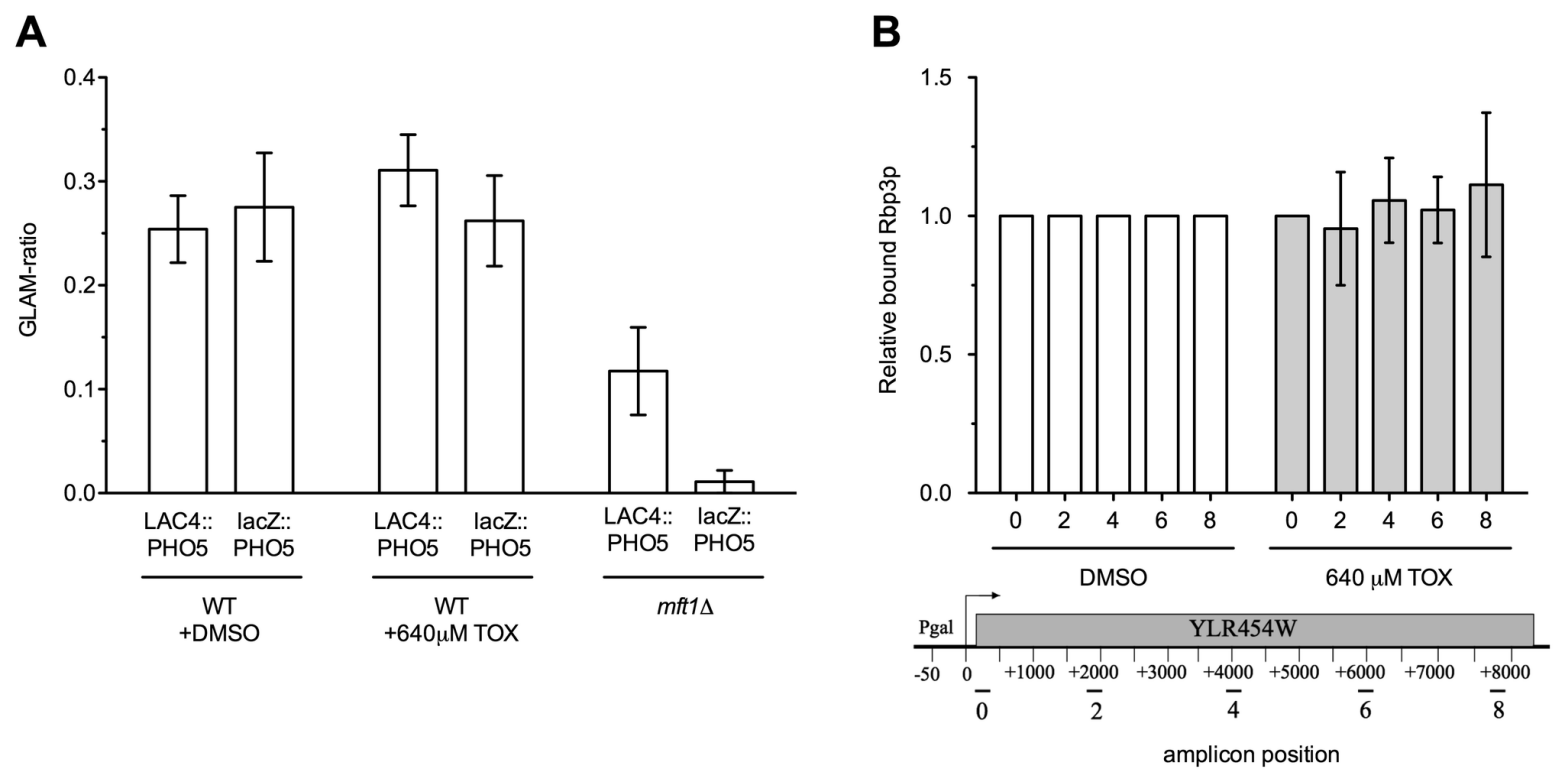
Toxaphene does not inhibit transcription elongation. (**A**) GLAM-ratios are not altered upon toxaphene treatment. The acid phosphatase activity of long (*PHO5-lacZ* or *PHO5-LAC4*) versus short (PHO5) transcriptional units was measured and the means and SE are shown for three independent experiments. The *mft1*Δ strain was used as a positive control. (**B**) Toxaphene does not affect RNA processivity. Levels of RNA polymerase II bound to different regions of a long gene were measured by chromatin immunoprecipitation.

We next examined toxaphene's potential to obstruct transcription elongation, reasoning that inhibition of this process could still occur via a mechanism distinct from that of MPA/6-AU. The GLAM assay has been developed to indirectly examine defects in transcription elongation [20], using the premise that mutants impaired in elongation are less able to transcribe long versus short transcription units. The toxaphene-sensitive transcription elongation mutants identified in this study have previously exhibited low scores when assayed for GLAM [20,25]. We measured the GLAM-ratios of acid phosphatase activity for a long (*PHO5-lacZ* or *PHO5-LAC4*) versus short transcription unit (*PHO5*) for BY4743 wild-type cells in the presence or absence of toxaphene, but did not find altered ratios upon toxaphene exposure (Figure 7A). Since GLAM is an indirect measurement of transcription elongation that may not recognize all transcription elongation defects, we directly assessed RNA polymerase II elongation by performing an RNA polymerase II processivity assay [15], where levels of RNA polymerase II bound to different regions of a long gene are measured. If transcription elongation were compromised under toxaphene treatment, the profile of RNA polymerase II would change in comparison to a non-treated sample, as seen in the case of 6AU and most transcription elongation mutants detected in this study [15]. However, the patterns of the toxaphene-treated and DMSO-treated samples were very similar, indicating that toxaphene does not affect RNA polymerase II processivity during transcription elongation (Figure 7B).

## Discussion

While the complex mixture of chlorinated terpenes known as toxaphene was once the most widely applied pesticide in the United States, its congeners are now considered persistent, bioaccumulative, and toxic. In 2004, these unfavorable characteristics resulted in toxaphene's addition to the Stockholm Convention on Persistent Organic Pollutants treaty as a member of the original “dirty dozen” compounds designated for international elimination or restriction. Those most at risk for exposure include Arctic populations who eat aquatic mammals and people consuming sport-caught fish from the Great Lakes [2]. Although toxaphene has been linked to diseases such as cancer and amyotrophic lateral sclerosis [4-7], cellular and molecular toxicity data associated with exposure are severely lacking. 

To discover potential mechanisms of toxicity, we screened the *S. cerevisiae* homozygous diploid non-essential deletion mutant collection to identify strains with altered growth in the presence of toxaphene. The majority of yeast genes confirmed as required for toxaphene tolerance are implicated in transcription elongation and associated processes (Figure 3), with additional resistance genes involved in nutrient utilization, drug transport, and various other cellular functions (Figures 4 and S1). Our results regarding nutrient utilization mutants are congruous with a report in which the toxicity of toxaphene was approximately 3-fold greater in rats fed a protein deficient diet [34]. Both results point to a need of a fully functional catabolic environment to resist toxaphene. Enrichment analyses indicated aerobic respiration mutants were sensitive to toxaphene (Table 2), which is consistent with toxaphene's ability to inhibit ATPases [31-33]. However, this group of strains was not studied to a large extent herein, as (1) the *qcr8*Δ strain (which has an electron transport chain defect) was a false positive (data not shown) and (2) most are petite and/or slow growing, which causes inherent competitive fitness defects during pool screens and may thus misinform further analyses. Future studies may elucidate the effects, if any, of toxaphene on aerobic respiration in yeast. The most sensitive strain identified by DSSA (and confirmed by growth curve assays) was *pdr5*Δ, which, not unexpectedly, lacks a drug resistance transporter involved in detoxification (Figure S1). Many yeast genes described in this study are conserved (Table 3) and may thus play a role in human toxicity. As the technical toxaphene mixture utilized in this investigation is comprised of hundreds of related chlorinated compounds, the congener(s) responsible for the observed toxic effects in yeast remain unknown. Moreover, both human metabolism and environmental weathering of toxaphene [3] may produce derivatives of differing toxicological relevance than those present in the technical mixture.

**Table 3:** Human orthologs of yeast genes required for toxaphene tolerance.

**Yeast gene**	**Human ortholog(s)**	**Human protein description**
*CDC73*	*CDC73*	Tumor suppressor; involved in transcriptional/post-transcriptional pathways
*DST1*	*TCEA1*	Transcription elongation factor A (SII)
*HXK2*	*HK2*	Hexokinase 2
*IRA2*	*NF1*	Neurofibromin 1, tumor suppressor
*PDR5*	*ABCG2*	ATP-binding cassette protein
*RAD6*	*UBE2A/UBE2B*	Ubiquitin-conjugating enzyme E2A
*RPB9*	*POLR2I*	DNA-directed RNA pol. II subunit *RPB9*
*RTF1*	*RTF1*	Role in transcription-coupled histone modification
*SAC3*	*GANP*	Synonym MCM3AP, minichromosome maintenance protein 3
*SPT4*	*SUPT4H1*	Regulates mRNA processing and transcription elongation by RNA pol. II
*STP1*	*ZNFN1A4*	Zinc finger protein, subfamily 1A, 4 (Eos)
*THO2*	*THOC2*	Component of the THO subcomplex of the TREX complex
*THP1*	*PCID2*	PCI domain containing 2
*TKL1*	*TKTL1/2, TKT*	Transketolase
*UBP3*	*USP10*	Ubiquitin specific peptidase 10

Deletion of any of these yeast genes caused sensitivity to toxaphene (listed in alphabetical order).

Although various -omics approaches have been utilized to examine the molecular effects of toxaphene, its mechanism(s) of toxicity remain ambiguous and findings directly related to transcription elongation have not been reported. Perhaps most relevant to this study is a report indicating that toxaphene altered expression of transcription termination genes in largemouth bass (Mehinto et al., in preparation). Woo and Yum [35] performed gene expression analyses in marine medaka, showing that toxaphene affected regulation of cytoskeletal, developmental, metabolic, nucleic acid/protein binding, and signal transduction genes. Increased expression of homocysteine methyltransferase, a zinc metalloenzyme involved in the metabolism of various amino acids [35], may indicate a link to the nutrient utilization genes described in this study (Figure 4). Toxaphene perturbed hepatic expression of one carbon metabolism and ribosomal biogenesis genes in adult male largemouth bass [36], while another study in the same organism could not establish changes in neuroendocrine signaling or neurotransmitter synthesis transcripts [37]. Proteomic analyses in the livers of largemouth bass identified differentially expressed proteins following toxaphene exposure, including an ion channel, a component of the pentose phosphate pathway, and a glutathione S-transferase [38]. Functional profiling in yeast has been performed with the related organochlorine pesticide dieldrin, but its toxic mechanism (altered leucine availability) is different from that of toxaphene [16].

Toxaphene-sensitive mutants also experience growth defects in MPA and 6-AU (for a comparison, see Table S3), two compounds that diminish transcription elongation by inhibiting GMP synthesis [26,27]. By decreasing nucleotide availability, MPA and 6-AU reduce RNA polymerase II elongation rate and processivity [15], with transcription elongation mutant sensitivity ascribed to the deleterious combination of transcriptional defects [39]. Although toxaphene and MPA synergistically inhibit yeast growth (Figure 5), two different elongation assays did not detect any effect of toxaphene on transcription elongation (Figure 7), unlike results seen with MPA and 6-AU [15]. Intriguingly, several toxaphene tolerance genes, such as *SAC3*, *SPT4*, *THP1*, and those encoding components of the THO complex, are associated with other processes tightly coupled to transcription elongation, such as mRNA processing, mRNA nuclear export, or transcription-coupled nucleotide excision repair [23,40-44]. The possibility that one of these cellular operations is required for toxaphene resistance led us to examine mRNA export in presence of toxaphene using *in situ* hybridization, however, our findings were inconclusive (data not shown). Alternatively, toxaphene sensitivity of transcription elongation mutants may be attributed to defective expression of yet to be defined toxaphene tolerance protein(s). This type of indirect effect occurs in elongation mutants exhibiting sensitivity to the nucleotide depleting compound 6-AU; these deletions cannot sufficiently induce Imd2p, the protein that combats 6-AU toxicity by restoring nucleotide levels [45].

By identifying mutants with altered growth in toxaphene, our study advances understanding of the genetic requirements for the toxaphene response in yeast. Despite evidence indicating transcription elongation mutants exhibit sensitivity to toxaphene, our data suggest toxaphene does not affect transcription elongation itself. Instead, we propose toxaphene tolerance requires a yet to be identified cellular process closely associated with transcription elongation, as toxaphene synergism with MPA indicates a likely effect along this pathway. Using data provided in this study, further pathway-specific investigations in yeast or humans may elucidate the distinct mechanism of toxaphene toxicity.

## Supporting Information

Figure S1 **Additional genes required for toxaphene tolerance.** The area under the curve (AUC) was calculated for strains treated with 640μM or 960μM toxaphene and expressed as a percentage of the AUC for the untreated strain. All bars represent the mean and SE for three independent cultures. (**A**) Mutants lacking drug resistance genes are sensitive to toxaphene. (**B**) Various mutants were confirmed to display sensitivity to toxaphene.
(TIF)

Table S1 **Primers utilized in transcription elongation assays.** Primer sequences are listed.
(PDF)

Table S2 **Mutants displaying altered growth in toxaphene.** DSSA identified strains sensitive or resistant to various doses of toxaphene. A negative log_2_ value signifies sensitivity at the corresponding dose, while a positive log_2_ value signifies resistance.
(XLS)

Table S3 **Mutants displaying sensitivity to both toxaphene and MPA, 6-AU, or 4-NQO.** A literature search was conducted to identify overlapping mutant sensitivities between toxaphene, the GMP synthesis inhibitors mycophenolic acid (MPA) and 6-azauracil (6-AU), and the model carcinogen 4-nitroquinoline-N-oxide (4-NQO).
(PDF)
