# Supplementary figures and images for: A Genome-Wide Screen Identifies Yeast Genes Required for Tolerance to Technical Toxaphene, an Organochlorinated Pesticide Mixture
Source: PLoS One. 2013 Nov 18;8(11):e81253. doi: 10.1371/journal.pone.0081253 (PMC3832591; doi:10.1371/journal.pone.0081253)

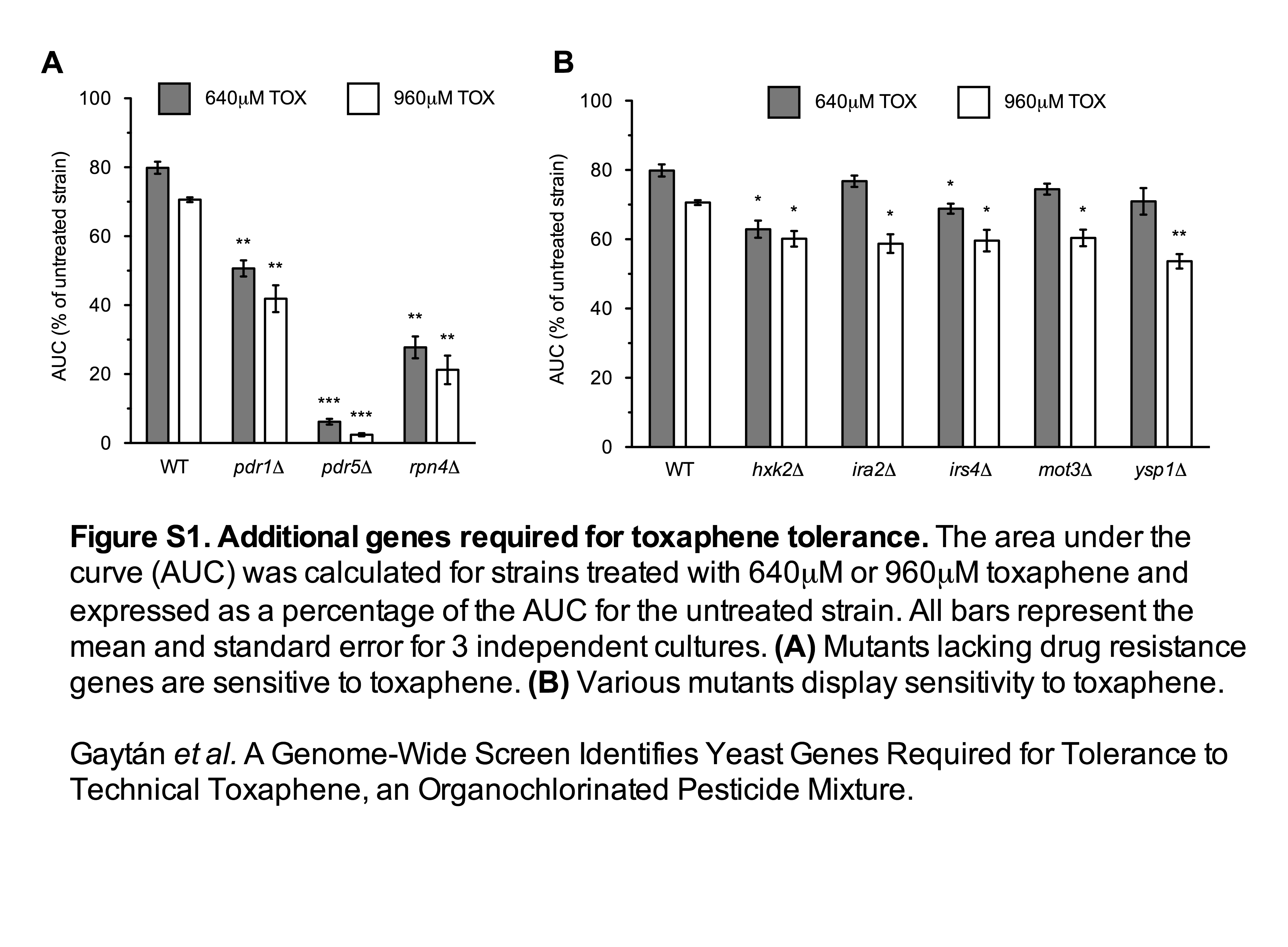

Supplement: Figure S1 — Additional genes required for toxaphene tolerance. The area under the curve (AUC) was calculated for strains treated with 640μM or 960μM toxaphene and expressed as a percentage of the AUC for the untreated strain. All bars represent the mean and SE for three independent cultures. (A) Mutants lacking drug resistance genes are sensitive to toxaphene. (B) Various mutants were confirmed to display sensitivity to toxaphene. (TIF) [file pone.0081253.s001.tif]
