# Supplementary material for: A Genome-Wide Screen Identifies Yeast Genes Required for Tolerance to Technical Toxaphene, an Organochlorinated Pesticide Mixture
Source: PLoS One. 2013 Nov 18;8(11):e81253. doi: 10.1371/journal.pone.0081253 (PMC3832591; doi:10.1371/journal.pone.0081253)
Supplement: Table S1 — Primers utilized in transcription elongation assays. Primer sequences are listed. (PDF) [file pone.0081253.s002.pdf]

Table S1. Oligos for quantitative PCR in chromatin immunoprecipitation experiments.

**Primer Name**	**Sequence**
1_100.txt-3F	GATGTTTCCGATTAATGTTCTACTGTACAA
1_100-66R	GCTCCATAAGAAAGTCACTGCAAA
1900-2000-3F	AGACAGAAGGAAATTTTACCAAGCG
1900-2000-63R	AATCGAAAAAATCAGGTAGTTGCTG
3800-4100-191F	GATATGCTTCAATCCGACAGAGAG
3800-4100-258R	TCAACAGTTACCGATGGTATTAAAGG
5800-6100-2F	AGCCGGACAAACAGAACAGC
5800-6100-71R	CAGGGTCTTTTTGGTGTTTTTCA
7600_7700.txt-21F	GTTGGACAATCTTAAAGTCGGGA
7600_7700.txt-92R	GTTGGACAATCTTAAAGTCGGGA
