# Supplementary material for: A Genome-Wide Screen Identifies Yeast Genes Required for Tolerance to Technical Toxaphene, an Organochlorinated Pesticide Mixture
Source: PLoS One. 2013 Nov 18;8(11):e81253. doi: 10.1371/journal.pone.0081253 (PMC3832591; doi:10.1371/journal.pone.0081253)
Supplement: Table S3 — Mutants displaying sensitivity to both toxaphene and MPA, 6-AU, or 4-NQO. A literature search was conducted to identify overlapping mutant sensitivities between toxaphene, the GMP synthesis inhibitors mycophenolic acid (MPA) and 6-azauracil (6-AU), and the model carcinogen 4-nitroquinoline-N-oxide (4-NQO). (PDF) [file pone.0081253.s004.pdf]

Table S3. Mutants displaying sensitivity to both toxaphene and MPA, 6-AU, or 4-NQO.

Transcription elongation mutants			
**Toxaphene-sensitive strain**	**Sensitivity to**		
	**MPA**	**6-AU**	**4-NQO**
*hpr1*Δ	2	2	
*mft1*Δ	7		
*tho2*Δ	4		4
*dst1*Δ	2, 4, 6, 7	2, 10	
*spt4*Δ	4	9	4
*cdc73*Δ	4		4
*rtf1*Δ	2, 4	2	
*spt20*Δ	3, 4	2	4
*sac3*Δ	2		
*thp1*Δ	10	10	
*ctk3*Δ	4	2	4
*rad6*Δ	2, 4		4
*rpb9*Δ	1, 4, 5		4
*snf6*Δ	2, 4	2	
*ubp3*Δ	7, 10	8, 10	
*mot3*Δ	2	2, 10	

Nutrient utilization mutants			
**Toxaphene-sensitive strain**	**Sensitivity to**		
	**MPA**	**6-AU**	**4-NQO**
*dal81*Δ	2, 7		
*stp1*Δ	2, 10	10	
*ure2*Δ	4		
*ira2*Δ	2		

A literature search was conducted to identify overlapping mutant sensitivities between toxaphene, the GMP synthesis inhibitors mycophenolic acid (MPA) and 6-azauracil (6-AU), and the model carcinogen 4-nitroquinoline-N-oxide (4-NQO). Numbers indicate the references cited below.

1. Betz, J. L., Chang, M., Washburn, T. M., Porter, S. E., Mueller, C. L., Jaehning, J. A. (2002). Phenotypic analysis of Paf1/RNA polymerase II complex mutations reveals connections to cell cycle regulation, protein synthesis, and lipid and nucleic acid metabolism. *Mol. Genet. Genomics*. **268**, 272–285.
2. Desmoucelles, C., Pinson, B., Saint-Marc, C., Daignan-Fornier, B. (2002). Screening the yeast "disruptome" for mutants affecting resistance to the immunosuppressive drug, mycophenolic acid. *J. Biol. Chem.* **277**, 27036–27044.
3. Dudley, A. M., Janse, D. M., Tanay, A., Shamir, R., Church, G. M. (2005). A global view of pleiotropy and phenotypically derived gene function in yeast. *Mol. Syst. Biol.* **1**, 2005.0001.
4. Gaillard, H., Wellinger, R. E., Aguilera, A. (2007). A new connection of mRNP biogenesis and export with transcription-coupled repair. *Nucleic Acids Res.* **35**, 3893–3906.
5. Gibney, P. A., Fries, T., Bailer, S. M., Morano, K. A. (2008). Rtr1 is the Saccharomyces cerevisiae homolog of a novel family of RNA polymerase II-binding proteins. *Eukaryotic Cell*. **7**, 938–948.
6. Gómez-Herreros, F., De Miguel-Jiménez, L., Morillo-Huesca, M., Delgado-Ramos, L., Muñoz-Centeno, M. C., Chávez, S. (2012). TFIIS is required for the balanced expression of the genes encoding ribosomal components under transcriptional stress. *Nucleic Acids Res.* **40**, 6508–6519.
7. Kapitzky, L., Beltrao, P., Berens, T. J., Gassner, N., Zhou, C., Wüster, A., Wu, J., Babu, M. M., Elledge, S. J., Toczyski, D., Lokey, R. S., Krogan, N. J. (2010). Cross-species chemogenomic profiling reveals evolutionarily conserved drug mode of action. *Mol. Syst. Biol.* **6**, 451.
8. McCullock, S., Kinard, T., McCullough, L., Formosa, T. (2006). blm3-1 is an allele of UBP3, a ubiquitin protease that appears to act during transcription of damaged DNA. *J. Mol. Biol.* **363**, 660–672.
9. Morillon, A., O'Sullivan, J., Azad, A., Proudfoot, N., Mellor, J. (2003). Regulation of elongating RNA polymerase II by forkhead transcription factors in yeast. *Science*. **300**, 492–495.
10. Riles, L., Shaw, R. J., Johnston, M., Reines, D. (2004). Large-scale screening of yeast mutants for sensitivity to the IMP dehydrogenase inhibitor 6-azauracil. *Yeast*. **21**, 241–248.
